# Value and feasibility of South-South Medical Elective Exchanges in Africa

**DOI:** 10.1186/s12909-020-02224-z

**Published:** 2020-09-21

**Authors:** Katy Daniels, Emma Thomson, Faith Nawagi, Maaike Flinkenflögel

**Affiliations:** 1 School of Medicine, University of Dundee, Dundee, UK; 2 College of Medicine, Blantyre, Malawi; 3 ECFMG®│FAIMER®- GEMx, Kampala, Uganda; 4 University of Rwanda, Kigali, Rwanda; 5 KIT (Royal Tropical Institute), Amsterdam, The Netherlands

**Keywords:** Medical electives, Global health, Low income countries, Student feedback, Evaluation

## Abstract

**Background:**

An elective is part of the curriculum where students have the flexibility to choose both the study topic and location. International medical electives are a well-established part of curricula at most medical schools in high-income countries. They are highly valued by students and have proven educational benefits, though do come with challenges, such as lack of reciprocity. Low and middle-income countries frequently host students from high-income countries providing learning opportunities, yet also carry the burden of supervision and resource consumption, whilst their students get few elective opportunities. This study explores the value and feasibility of South-South Medical Elective Exchanges (SSMEE), which creates elective opportunities for African medical students in other African countries to create reciprocity within the elective system.

**Method:**

A qualitative evaluation of the South-South Medical Elective Exchanges was conducted using a case study approach. Four African medical schools, College of Medicine, Malawi; University of Rwanda, Rwanda; University of Witwatersrand, South Africa and Makerere University, Uganda participated in the pilot study in 2017/18. Each institution selected two students to participate in a four-week elective to another participating institute. Participating students completed a pre-elective questionnaire and a post-elective interview exploring expectations, learning outcomes, challenges and how they are applying this learning. Data was analysed thematically.

**Results:**

Data presented is from six of the eight participating students. All students found the elective a valuable experience and learning was demonstrated in four key areas: clinical knowledge and skills; attitudes; personal and professional development and global perspectives. For some, it challenged their assumptions of what an elective is because valuable learning can be achieved whilst remaining in Africa. The main challenge found related to funding the elective.

**Conclusions:**

The SSMEE model is feasible and provides valuable learning for participating students and their peers/colleagues. Financing electives remains the biggest challenge. Since this pilot study, SSMEE has become part of a regional elective exchange network in Africa with an additional four institutions in three other countries. As such SSMEE has resulted in increased opportunities for African medical students and better educational outcomes that are likely to have a positive effect on healthcare systems in Africa.

## Background

Global health issues, the need to understand health disparities and equip students appropriately are increasingly relevant [1] in light of globalisation. As such, educators have a responsibility to prepare students for such challenges and electives can help achieve this.

**Fig. 1:**
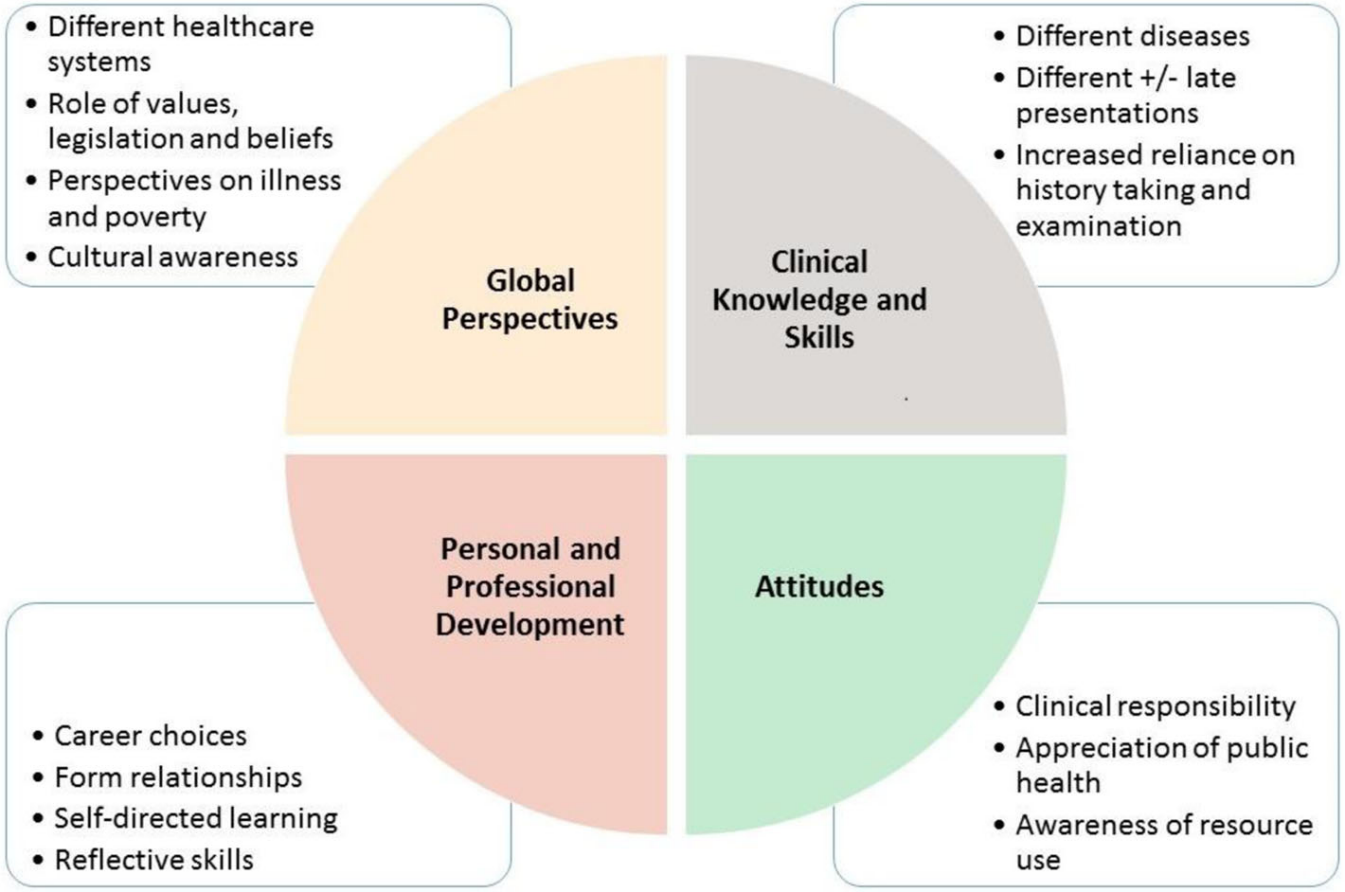
Value of clinical electives for medical students

An elective is part of the curriculum where students choose both study topic and location, often in another country. International medical electives (IME) are well-established within curricula at most medical schools in high-income countries (HIC) [2–4] because of the learning students gain, which broadly includes: clinical knowledge and skills, attitudes, personal and professional development and global perspectives [5–8] (Fig. 1). They also provide significant transformative experiences for many and reignite the vocational drive that led many to enter the medical profession [9], whilst potentially recruiting trainees to primary care and underserved areas [8].

Of British students undertaking an IME approximately 40% of them choose a developing country [3]. In Australia, this can be as high as 59% [4]. Based on medical electives alone, universities and healthcare institutions in Africa receive thousands of students yearly from HIC [10].

Increasing consideration is being given to the ethics of electives, including the positive and negative consequences for host communities [5, 9]. International students undertaking electives can benefit the host institution by enhancing the host’s reputation [5, 11, 12]; bringing opportunities for international training for local staff, equipment donation and financial gain [5, 12]; bringing international collaboration opportunities [11], and providing clinical services [11, 13] which sometimes fills gaps in healthcare provision [11]. Their presence enriches the educational environment due to varied backgrounds [1], allowing the exchange of ideas and experiences [11]. In this setting, learning can become bidirectional between host staff and elective students [13]. Elective students can increase supervisors’ motivation to continue professional learning [11]. Finally, some hosting institutions see elective students as a potential source of future staff [13].

However, students undertaking IME in under-resourced settings potentially exacerbate the lack of resources [5, 11] and consume staff time through teaching, supervision and translation, which could otherwise be used for other activities [14]. Such electives can also include cultural voyeurism and replicate colonialist practices, promoting the ‘West knows best’ attitude, cultural insensitivities and power imbalance [7, 15]. There is also the risk of harm to patients due to language barriers, lack of cultural competency [11] and uninformed consent [16]. As such, host communities in low-middle income countries (LMIC) often experience significant burdens.

An awareness of the need for change exists [5, 7] with sending institutions, government reports [17] and importantly, elective host communities in LMICs [11, 15, 16] recognising that reciprocity should be increased in current elective systems. Students in LMIC rarely get such elective opportunities according to the staff at the medical schools participating in this research. We expect that African medical students have as much to gain from studying internationally as their HIC colleagues, and there is some evidence consistent with this [10, 18]. However, there are numerous constraining factors for IME for African students including limited funding, visa restrictions and the lack of organisational support [10].

Some elective opportunities exist, through institutional partnerships with bilateral exchange programmes, where LMIC elective host institutions are given elective opportunities in other countries, typically HIC [19, 20]. However, they are limited in number and organising them on a larger scale is difficult and costly. The International Federation of Medical Students Associations (IFMSA) ‘SCOPE’ programme creates elective opportunities by providing bilateral exchanges using a network of locally and internationally active students in both HIC and LMIC [21, 22]. However, costs such as exchange fees and international travel make even these programmes unaffordable for the majority of students in LMIC.

South-South Medical Elective Exchanges (SSMEE) is an alternative elective model that aims to create IME opportunities for African medical students in another African country for several reasons. Firstly, students should experience healthcare systems similar enough to their own that learning will be transferable back to their own context, secondly, such electives have a reduced risk of brain-drain and thirdly costs could be lower than an elective further afield, which would increase financial sustainability.

The concept of SSMEE was presented at the 2015 *The Network: Towards Unity for Health* conference in South Africa. This international conference was an opportunity to share the concept to gauge existing interest in SSMEE. Several African medical schools expressed interest so a pilot study with four African medical schools (Malawi, Rwanda, South Africa, and Uganda) was developed.

Despite the evidence of the value of electives for students from HIC, there remains little evidence for undergraduate students in LMIC. The evaluation of SSMEE aimed to explore how South-South medical electives are valuable and/or feasible in sub-Saharan Africa.

## Methods

A qualitative case study approach was used to gain a detailed understanding of the SSMEE pilot through evaluation [23]. Being an instrumental case [24] it sought to gain insight into specific issues and problems in one bounded case to determine how SSMEE was valuable and feasible.

**Fig. 2:**
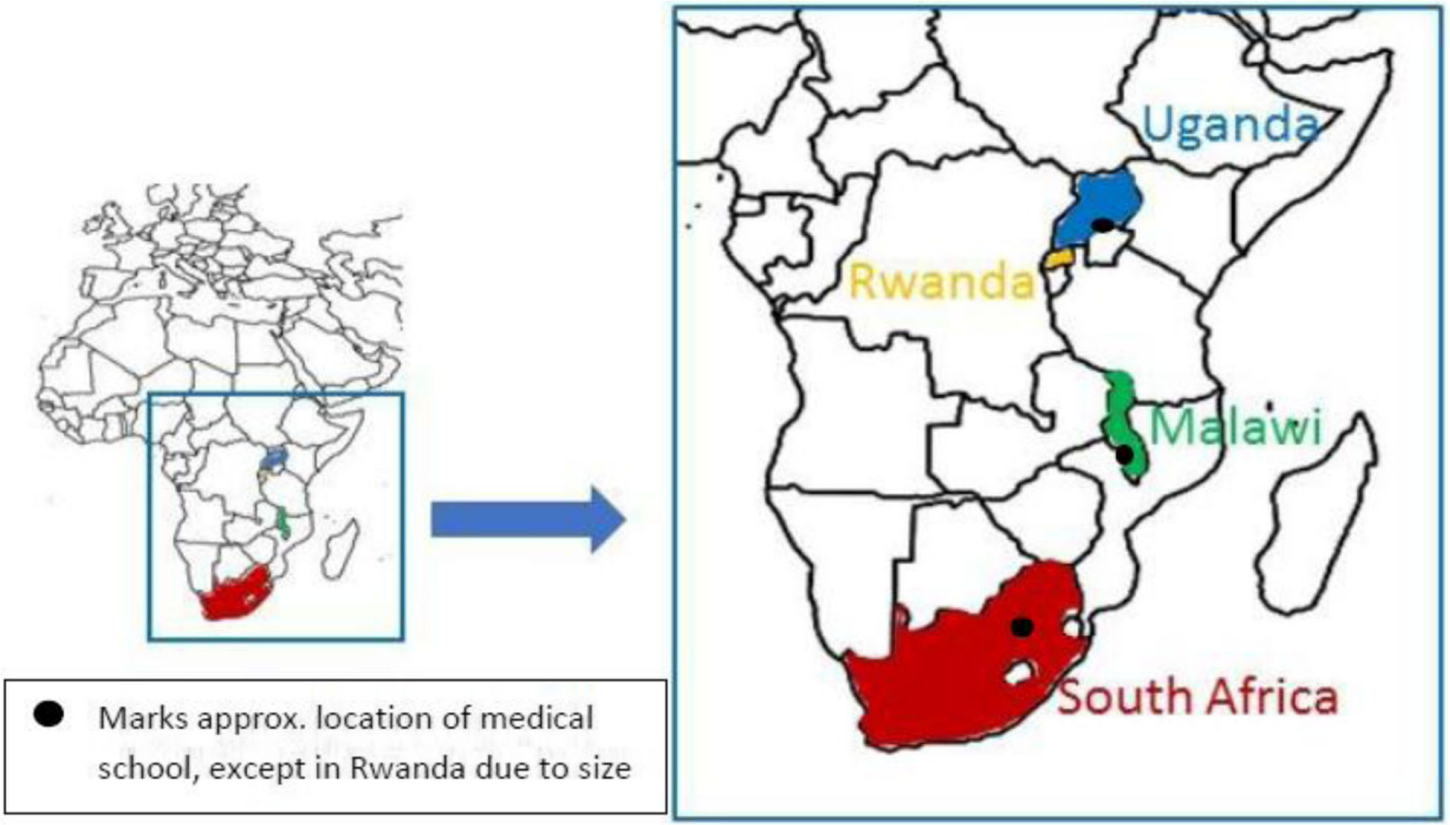
Map with the four participating countries

The subjects were eight final year medical students, selected by their respective medical schools to participate in SSMEE, and composed of two students each from the College of Medicine, Malawi; University of Rwanda, Rwanda; University of Witwatersrand, South Africa and Makerere University, Uganda (Fig. 2). Students were selected by their grades and statements of why they should be able to participate. Any student that had already undertaken an international elective was excluded. The organisation of this pilot study was supported by the Global Educational Exchange in Medicine and the Health Professions (GEMx) program, a service of Educational Commission for Foreign Medical Graduates’ and the Foundation of Advancement in Medical Education (ECFMG®│FAIMER®) [25]. GEMx provided the web-based elective application platform that allowed the centralisation and operationalisation of the student electives, a coordination center to oversee activities and mobility of the students, as well as a 1000USD grant for each student as they went on electives to help reduce costs. Students were required to register with the relevant Medical School, as per other elective students, and where registration with a healthcare or medical governing body was required, support was provided for this process. Students were advised that SSMEE was part of a research project and so they would be invited to participate in the evaluation of the programme in line with the good ethical practice that involved gaining of written informed consent [26].

### Data collection

Electives took place between June 2017 and January 2018 and were of 4 weeks duration. Two methods of data collection were used: firstly, a pre-elective questionnaire (Additional file 1) and secondly a post-elective semi-structured interview (Additional file 2). Both were completed in English.

The questionnaire obtained helpful background information regarding the student, previous travel, why they wanted to participate, desired learning objectives and any concerns they had about the elective. The questionnaire was administered via email and completed before the elective.

Semi-structured interviews were conducted within a few weeks of the elective to allow the researcher to evaluate participants’ learning and gather information about their experiences whilst enabling clarification, further questioning and probing of answers [27]. With participants in four African countries and the interviewer (KD) in Scotland, face-to-face interviews were not feasible. Anticipated internet challenges discounted internet-based interviews and so interviews were conducted using WhatsApp instant messenger. The credibility of responses was sought by giving participants control of the interview such as selecting the time of the interview, the ability to leave the interview and come back to it if required, putting them at ease and then giving an invitation to share their thoughts [28]. The transcripts were downloaded and anonymised.

### Data analysis

Framework analysis [29] was undertaken of both questionnaire and interview data. Inductive analysis was used following the six steps described by Braun & Clarke [28]: familiarising oneself with the data, generating initial codes, searching for the themes, involved reviewing of the themes, defining and naming the themes and finally, producing the report. Two of the six interview transcripts were analysed and coded independently by a second analyst for triangulation, then as a thematic framework was constructed, discussion of coding and discrepancies took place between analysts thus adding rigour to the analysis.

The trustworthiness of the research was considered. The use of two methods of data collection and the addition of a second analyst provided triangulation and improved credibility, while the detailed description of the results aided transferability. In addition, reflexivity was practiced to identify data that might have been over or underrepresented due to personal preconceptions or biases; or data that did not fit within patterns identified as a means of enhancing confirmability.

Ethical approval was granted by research ethics committees at the University of Dundee, Scotland, College of Medicine, Malawi, the University of Rwanda and the University of Witwatersrand, South Africa. Local ethical approval was not required in Uganda.

## Results

All eight medical students in SSMEE agreed to participate in the research. However, the two students from the University of Rwanda had not undertaken their elective within the intended time frame due to delayed student selection by the university and restrictions as to when their elective could take place within the academic calendar. As such, data from six of the eight students is presented, each identified by a letter from A-F. The six students undertook an elective in Malawi, Rwanda or South Africa, however, the elective destination is not specified as this could breach student confidentiality.

### Value of the elective

Interview data highlighted the perceived value for students, which has been grouped into four categories: clinical knowledge and skills, personal and professional development, attitudes and global perspectives. Although the data was independently analysed, the same themes emerged as presented in Fig. 1 [5–8] hence the same themes were used.

#### Clinical knowledge and skills

All six students described the elective as providing opportunities to apply and develop their clinical knowledge and skills, such as: history taking and examination skills, communication skills, diagnostic capabilities, and practical procedure skills. Sometimes this was by exposure to diseases, such as schistosomiasis and malaria not encountered in their own hospital setting and in other cases it was the application of previous learning. Some students felt more independent in patient management, such as examination of the neurological system, auscultation of cardiac murmurs and placing central lines, chest drains, using sonography for trauma assessment and use of a defibrillator, not readily available in their own institution. An example of this type of learning is:

“I was able to see and interpret MRI images something I hadn’t had adequate opportunity to do at my college … ..picked up some techniques they used while operating … .improve my capabilities in taking a focused history and what to look for as we examine patients depending on the working diagnosis” (A)

This elective also led some to reflect on differences between their elective experience and how things were done in their home institution as well as the application of their learning:

“I realised that patients were being explained everything and being involved in every step of the way including them to write in the file what they had understood and sign against it. I found this very striking since back home, we mostly talk to rather than talking with the patients. I am going to practice this and share it with my colleagues” (B)

“I had a patient with a severe head injury [in home country], and I used the principles that I had been practising in [elective country]” (F)

All students felt their learning was applicable to their home context.

#### Personal and professional development

The elective resulted in students’ personal and professional development in areas such as vocational drive, meeting their own goals and improving confidence:

“In terms of myself, it reaffirmed me that I was in the right profession and that I was becoming the competent doctor I've always wanted to be... Before the elective, I didn't think I'd be comparable with a student in a different institution but now I know I can be and there's no excuse for not dreaming big.” (F)

#### Attitudes

Personal attitudes were challenged resulting in a desire to change, e.g. be kinder to patients or less wasteful of resources.

Several students mentioned they would share what they learned with peers/colleagues, therefore the benefits of electives go further than just the individual students.

“I am advantaged compared to my peers to know some more but I also share with them the ideas and knowledge.” (A)

#### Global perspectives

Students experienced different languages, cultures, healthcare systems, beliefs/values and diet allowing them to reflect how this impacts local populations and look at their own background and healthcare system from another perspective.

“The culture had a toll on health-seeking behaviour. Most of the patients believe in traditional medicine and therefore would visit traditional healers and only come to hospital when everything failed, therefore they would present late to hospital!” (A)

Students recognised that teaching methods vary between countries and highlighted their positive experiences of elective-based teaching:

“I observed that the professors and lecturers (at least the ones that taught me) are not intimidating and always want to teach students. It is somehow different to [home country] where sometimes you feel intimidated.” (E)

### Feasibility

The feasibility of SSMEE was explored by considering student’s perceptions of an IME in another African country and the challenges they anticipated or encountered.

### Student perceptions

Students described their feelings about IME in another African country. Initial attitudes among the six students varied, with some changing over time. Students A and B were happy to have the opportunity to go anywhere. Students D and E initially preferred to go out of Africa. With hindsight, however, student E recognised the elective undertaken provided him/her the opportunity to see something different, as s/he had desired, and student D is now motivated to visit more African countries rather than leave Africa because “there is a lot more to learn especially in Africa with regards to resource constraints and the burden of disease.” Student C, also favoured the elective within Africa:

1. “The disease burden within Africa is similar, …, by staying here you are learning similar things just in a different way and different place.
2. From my understanding, African countries have more hands-on learning opportunities because medical students are more hands-on.
3. Charity begins at home, so why travel across an entire ocean for something you can achieve in your own backyard.
4. Africans should build and support other Africans, and this is a good way to do that.”

Student F initially associated ‘elective’ with leaving Africa, however, had “such an amazing experience … can’t imagine having the experience I had in an outside [of Africa] country”.

### Challenges anticipated or encountered

In the pre-elective questionnaire, five of the six students identified potential concerns such as: getting lost, communication, cultural awareness, the potential for culture shock, insufficient personal knowledge and skills, safety and money shortages or non-functional bank cards. Actual challenges encountered were discussed in post-elective interviews:

#### Finance

Each student received a 1000USD bursary towards elective expenses. Students were asked how much their elective cost and five of the six students provided figures. Costs ranged from 900USD to 1600USD. At least four out of six students required further funding. Four of the six students supplemented the bursary with their own finance obtained via family or friends. Only two students had additional costs covered by their own University. For those covering their own additional costs, limitations of funds had a considerable impact:

“I mainly faced a shortage of funds. I had no stipend to cater for meals especially in the last week. Most of the money for upkeep I got from home and it was not really enough.” (A)

Student D described few opportunities to pay by bank card in their elective country resulting in high bank charges to withdraw money which could have been avoided had they known to expect this.

#### Language barrier

The language barrier was a concern expressed by students prior to their elective. Two students revisited the language barrier during post elective interviews. One student mentioned that discussions took place in English and the [local] language was easy to learn. Student D however found:

“It was quite challenging to talk to patients and examine because of the language barrier. I had to learn some [local language] words to ease the consultation. I also asked for help from one of the doctors or nurses to translate” (D)

Student D declared s/he would increase efforts to learn different languages used in his/her country to improve communication with patients.

#### Practical issues

A range of practical issues were encountered by students. South African students wanted to travel to Rwanda however were unable to due to political reasons. Another student encountered visa problems because the allowed time was inadvertently overstayed.

Student E encountered logistical challenges because the accommodation was not as close to the hospital as expected, incurring transport costs. Other unexpected issues arose in terms of electricity supply and supervision:

“I was not aware about the electricity cuts. I would have been better prepared for them.”(D)

Concerns over safety and theft had been expressed pre-elective by student E although neither was experienced by any of the students. Student C shared that s/he had been concerned about the possibility of xenophobia, however, this was not encountered and instead experienced positive social interactions.

## Discussion

### The south-south medical elective exchange model

One of the criticisms of existing elective models, when students go from HIC to LMIC is the ‘one way’ nature of electives [5] with hosting LMIC’s not being equal beneficiaries in the process. SSMEE moved away from this ‘one way’ nature with participating institutions enrolling equal numbers of students, providing equal opportunities for all institutions and hence achieving reciprocity. In this sense, SSMEE was similar to the IFMSA bilateral exchange model [21].

When this project commenced the South-South exchange model was not documented in the literature for undergraduate students, though an example for postgraduate family medicine trainees, going from Rwanda to South Africa, [10] demonstrated positive learning outcomes. Our study showed that the South-South model is valuable and feasible for undergraduate students and that multiple LMIC can be involved in sending and hosting students. Involving multiple countries in SSMEE, rather than specific partnerships [19, 20], importantly creates choice for participating students, an important element of an elective [9].

The authors wanted to know how the opportunity for an African elective would be perceived in comparison to an elective outside Africa. The spectrum of opinions given before the elective was wide, from preferring to travel out of Africa to wanting to remain. However, the existing preconceptions were challenged and all students spoke highly of their African elective experience. Although staying within Africa might not be the preferred option for all, SSMEE provides additional opportunities and choices, important factors for electives [9]. SSMEE also allow students to learn with and from their African counterparts which hopefully will encourage participants to remain within Africa.

### The value of SSMEE

The learning gained from SSMEE can be categorised according to the four domains previously described by Dowell and Merrylees: clinical knowledge and skills; attitudes; personal and professional development and global perspectives [5]. Currently, the recognised benefits of IME’s are largely based on data from students studying in HIC [5, 6, 8] however this study has shown that these benefits also apply for medical students in LMIC going to another LMIC.

The authors speculated that learning in another African country would be transferable to the student’s own learning environment which was confirmed during post-elective interviews. Students described the application of their learning and behaviour changes, albeit self-assessed, which are important outcomes of learning.

An additional gain described in this study, not mentioned in existing literature was the exposure to alternative teaching methods. It could be expected that this can encourage one to reconsider their own education system through different lenses.

Attitude changes can occur through an IME [5, 13, 30, 31] and similar attitudinal changes were identified in SSMEE participants. This study provides evidence that such learning can be gained within Africa and students often share this learning with others, spreading the benefits further afield. Therefore, students do not need to travel as far as HIC to gain this learning, which could reduce costs, a recognised barrier to electives [9, 32, 33].

### Challenges

The SSMEE pilot study identified challenges discussed under the themes of finance, harm to patients and practical issues.

### Finance

The most significant challenge is the funding of electives. With elective costs a potential challenge for students in HIC [34, 35] it is unsurprising this was also an issue for SSMEE participants from LMIC. With the cost of a four-week elective exceeding the 1000USD grant in most instances, covering costs beyond this was a challenge for the students involved. Other sources of funding or bursaries are often not available to African medical students. Apart from the one university covering additional costs for their two students, other students did not have access to other bursaries. Addressing the financial costs of an elective remains the biggest hurdle for SSMEE and without the grant offered by GEMx programme financing the elective would have been a bigger hurdle for students. Though SSMEE will cost less, due to lower travel costs and lower living expenses, and so is a more financially viable and sustainable option if the desire is to create international elective opportunities with reciprocity.

### Practical issues

A few minor practical challenges were encountered by students, some of which might have been prevented with more pre-departure information and better planning on the part of students.

One student had expressed concern pre-elective of encountering potential hostile attitudes, such as xenophobia, in elective host countries. Although this was not encountered it does highlight the need to consider equity and diversity issues with respect to electives.

### Strengths and limitations of the study

The small size of the study could be seen as a limitation, however, being a pilot study it was intentionally small aiming to explore the value and feasibility of the model. Financial support was another key reason for the study size as student bursaries were only available for two students from each institution at this stage*.* Despite being small, the study has involved four different medical schools, each in a different country and with their own curricula and timetable demonstrating the feasibility of a multicentre model.

Selection bias may have occurred as participating institutions were self-selected and the institutions designated the students to participate in the electives, only excluding students who previously did an international elective. This selection process might have resulted in potential positive bias due to the positive attitudes towards the study from the outset. Additionally, this research project provided participants with an IME that they otherwise would not have had, which means they may have exaggerated the benefits of their elective, as a sign of gratitude.

## Conclusion

The SSMEE model provides valuable learning for participating students and their peers/colleagues. This pilot study has shown that the SSMEE model is feasible with four countries participating. Financing electives remains the biggest challenge. Since this pilot study in 2017/18, SSMEE has continued and has now merged with another GEMx project forming a regional elective exchange network in Africa with an additional four institutions in three other countries. More funding through GEMx, has been given to each institution to help offset elective costs. As such SSMEE has resulted in increased opportunities for African medical students and better educational outcomes that are likely to have a positive effect on healthcare systems in Africa. The value for host institutions and impact on patients has not been explored during this study and would warrant further research.

Although this pilot study was conducted long before the Covid-19 pandemic, this pandemic has highlighted the need for global health learning, restricted the ability to travel and raised awareness of the environmental impact of worldwide travel. As such, the need for sustainable elective models are more relevant now than ever. The effects of Covid-19 on medical electives, and specifically regional electives, would merit further study.

## Supplementary information

**Additional file 1.** What is the feasibility and value of South-South Medical Elective Exchanges in Africa Medical Student Pre-Elective Questionnaire.

**Additional file 2.** Medical Student Interview Schedule.

## Data Availability

The data set generated in this study is not publicly available as participant consent was not given for this and ethical approval was given for researchers only to have access to the data.

## Abbreviations

IME: International medical electives
SSMEE: South-South Medical Elective Exchanges
HIC: High-income countries
LMIC: Low-middle income countries
IFMSA: The International Federation of Medical Students Associations
GEMx: Global Educational Exchange in Medicine and the Health Professions
ECFMG®│FAIMER®: Educational Commission for Foreign Medical Graduates’ and the Foundation of Advancement in Medical Education
USD: United States Dollar
