# Supplementary material for: Value and feasibility of South-South Medical Elective Exchanges in Africa
Source: BMC Med Educ. 2020 Sep 21;20:319. doi: 10.1186/s12909-020-02224-z (PMC7503442; doi:10.1186/s12909-020-02224-z)
Supplement: Supplementary file 1 — Additional file 1. What is the feasibility and value of South-South Medical Elective Exchanges in Africa Medical Student Pre-Elective Questionnaire. [file 12909_2020_2224_MOESM1_ESM.docx]

**Appendix 1: What is the feasibility and value of South-South Medical Elective** **Exchanges in Africa Medical Student Pre-Elective Questionnaire**

1. Gender: Male/ Female/ Do not wish to say

2. Which year of study are you in?

3. What country are you studying in?

4. Have you ever been abroad before? If so, where and why?

5. What country are you going to for your elective?

6. What department/s are you intending to be working with?

7. Why do you want to participate in this exchange programme?

8. Complete the following table with up to six things that you hope to gain from this international medical elective. Then rank each one in order of how important it is to you to achieve with 1 = most important and 6=least important.

| What do you hope to gain | Ranking |
| --- | --- |
|  |  |
|  |  |
|  |  |
|  |  |
|  |  |
|  |  |

9. Is there anything you are worried about when going abroad?

Please note if you are worried about anything you may wish to speak to someone about this such as a member of staff at your university.

Thanks for your time!
