# Supplementary material for: Value and feasibility of South-South Medical Elective Exchanges in Africa
Source: BMC Med Educ. 2020 Sep 21;20:319. doi: 10.1186/s12909-020-02224-z (PMC7503442; doi:10.1186/s12909-020-02224-z)
Supplement: Supplementary file 2 — Additional file 2. Medical Student Interview Schedule. [file 12909_2020_2224_MOESM2_ESM.docx]

**Appendix 2: Medical Student Interview Schedule**

1. Introductions.

2. Explain purpose of interview and ensure have consent.

3. Why did you want to participate in this exchange programme?

4. Have you previously undertaken a clinical attachment/medical elective out with the country you are studying in?

- If yes: Was this optional or part of curriculum?
- How was this arranged?
- How was it funded?
- If no: Why not?

5. This elective has been part of a South-South exchange programme. Would you have rather gone out of Africa? Why?

6. Did you feel prepared for your international medical elective?

- If yes, how did you prepare?
- If no, with hindsight what would have helped you to feel more prepared?

7. What has been the value of this international medical elective?

- Can you give me any examples?

8. How is this learning relevant to you now you are back?

•Can you give me any examples?

•Will you do anything differently now?

•Can you explain how this IME has changed you?

9. How did your experiences compare to your expectations? (Interviewer will be able to refer back to pre-elective questionnaire and share content with interviewee)

•how do you feel about this?

•Is there overlap, if not, why?

10. Did you encounter any challenges?

•In the application process

•During the elective

•On returning home

11. How much did your elective cost, approximately?

•Travel, visa, accommodation, living expenses, hospital administration fee

12. We would like some feedback about how you found using the GEMx system for applying for your elective. Can you rate each of the following statements (scale will be spoken)

|  | Strongly disagree | Disagree | Uncertain | Agree | Strongly agree |
| --- | --- | --- | --- | --- | --- |
| The GEMx system was easy to use |  |  |  |  |  |
| There was an appropriate amount of information provided about the application process |  |  |  |  |  |
| There was an appropriate amount of information provided about the elective host institution (university/hospital) |  |  |  |  |  |
| There was an appropriate amount of information provided about the placement options |  |  |  |  |  |
| The application process ran smoothly |  |  |  |  |  |

13. Is there anything you would do to improve the GEMx system?

14. Is there anything else that you would like to share that you think would be helpful in evaluating this exchange programme?
